# Insights on *Juniperus phoenicea* Essential Oil as Potential Anti-Proliferative, Anti-Tyrosinase, and Antioxidant Candidate

**DOI:** 10.3390/molecules28227547

**Published:** 2023-11-11

**Authors:** Rim Ben Mansour, Hanen Wasli, Soumaya Bourgou, Saber Khamessi, Riadh Ksouri, Wided Megdiche-Ksouri, Susana M. Cardoso

**Affiliations:** 1 Laboratory of Aromatic and Medicinal Plants, Center of Biotechnology of Borj-Cedria, BP 901, Hammam-Lif 2050, Tunisia; rim.bmansour@gmail.com (R.B.M.); hanenwasli@gmail.com (H.W.); bourgousoumaya@yahoo.com (S.B.); sabirouch2004@yahoo.fr (S.K.); ksouririadh@gmail.com (R.K.); ksouriwided@yahoo.fr (W.M.-K.); 2 Faculty of Sciences of Tunis, University of Tunis El Manar, Tunis 1068, Tunisia; 3 Department of Education and Teaching, Higher Institute of Applied Studies in Humanities of Tozeur, Campus University, University of Gafsa, Gafsa 2100, Tunisia; 4 LAQV-REQUIMTE, Department of Chemistry, University of Aveiro, 3810-193 Aveiro, Portugal

**Keywords:** *Juniperus phoenicea*, North Tunisia, essential oil, GC-MS, anticancer, anti-tyrosinase

## Abstract

In this study, the anti-cancer, anti-tyrosinase, and antioxidant activities of essential oils (EOs) of berries and leaves of *Juniperus phoenicea* grown wild in North of Tunisia were investigated. The EO yields from leaves and berries were 1.69% and 0.45%, respectively. GC-MS analysis revealed that α-pinene is the predominant component in both EOs (44.17 and 83.56%, respectively). Leaves essential oil presented high levels of β-phellandrene (18%) and camphene (15%). The EOs displayed cytotoxic effects against MCF-7 breast cancer cell, HT-29 colon cancer, and the normal cells H9C2 cardiomyoblasts. Leaves oil strongly inhibited colon cell line proliferation (IC_50_ of 38 µg/mL), while berries essential oil was more potent against breast cancerous cells MCF-7 (IC_50_ of 60 µg/mL). Interestingly, berries essential oil exhibited high ability to inhibit melanin synthesis by inhibiting enzyme mono and diphenolase activities. Overall, the results suggested that the two oils are significant sources of healthy natural chemicals.

## 1. Introduction

Plants produce a remarkable set of secondary metabolites (SMs) with alleged health benefits, which form the basis of herbal remedies and even of pharmaceutical drugs. One of such examples is essential oils (EOs), i.e., complex mixtures of volatile compounds, which in many cases can be applied industrially for therapeutic purposes and food applications [1,2,3,4]. Consistent with this, different authors reported that EOs have high curative characteristics and less side effects than chemical drugs, acting through the modulation of oxidative stress and the targeting of inflammatory parameters. Additionally, the anticancer potential of EOs has been the focus of attention of many researchers, with many of them being claimed to suppress cancer. In this topic, EOs components, especially monoterpenes, have been claimed to exert multiple pharmacological effects on mevalonate metabolism, which could account for the tumor suppressive activity by terpenes [5]. Amongst them, monoterpenes alcohols were previously demonstrated to elicit a drastic reduction in the expression of two enzymes related to 5-fluorouracil (i.e., a widely used anticancer drug) cytotoxicity, namely thymidylate synthase and thymidine kinase. The first represents a crucial target for 5-fluorouracil in the de novo pathway of pyrimidine synthesis, which in turn is necessary for DNA synthesis, and thymidine kinase plays a key role in the complementary or alternative salvage pathway of pyrimidine synthesis in acute or pathological tissue stress. A malignant tumor of melanocytes is responsible for the majority skin-cancer-related deaths [6]. There is a current effort to search for tyrosinase inhibitors from nature through various approaches. Researchers are also focused on developing strategies for synergistic effects of multiple inhibitors and simultaneously regulating multiple targets to treat cancer [7]. Melanin polymers are high-molecular-weight pigments known for protecting the skin against the deleterious effects of UV rays. Many natural tyrosinase inhibitors, such as kojic acid, azelaic acid, hydroquinone, arbutin, and numerous phenolic compounds, are prospectively safer, more efficacious treatments for malignant neoplasms and can reduce excessive melanin production [8]. In this same context, plant EOs have attracted great interest as therapeutic active compounds and have been explored as inhibitors of tyrosinase [9,10]. In line with this, when evaluating the anti-tyrosinase of distinct medicinal herbs and food plants EOs, Aumeeruddy-Elalfi et al. [11] reported promising effects, comparable to kojic acid. In addition, due to their anti-melanogenic [9] and anti-aging properties [12], EOs also have various cosmetic applications and the potential to be used as a whitening agent or a plant-based therapy for skin darkening [13]. In this context, the ability of some EOs to inhibit tyrosinase, the enzyme implicated in the production of melanin in human skin, is of utmost importance, as this is involved in epidermal hyperpigmentation, which results in a variety of dermatological conditions like melisma, freckles, and age spots [14]. Also related to this, it is notable that nowadays, such potential applications can be empowered through open innovation approaches using digital communication platforms, which make it possible to contact huge audiences of relevant stakeholders swiftly and easily [15]. In fact, the advancement of digital technology and the emergence of open innovation methodologies have made it possible to establish a variety of virtual organizations and businesses that primarily coordinate their operations online [16]. International Natural Product Sciences Taskforce (INPST) is one of such open innovation platforms, aiming to bring together individuals and organizations interested in natural product scientific research and to facilitate their interactions by digital communication tools.

*Juniperus phoenicea* L., also known as red juniper, is distributed in Mediterranean countries of North Africa, extending to the Arabian coast of the Red Sea in the east and to the Canary Islands and Madeira in the west. The species’ wide geographic distribution allows for a tremendous amount of genetic variability [17]. In folk medicine, this plant is considered a remedy that is commonly used in many countries for the treatment of diarrhea, bronchitis, rheumatism, acute gonococcal infection, eczema, hemorrhoids, dysmenorrheal, sunstroke, and depurative disinfectant [18,19]. The leaves of *J. phoenicia* are used against bronco-pulmonary disease and as a diuretic, whereas the berries are used to calm the crises of all types of coughs and as an oral hypoglycemic agent [20]. Furthermore, the mixture of *J. phoenicia* leaves and berries is used as an oral hypoglycemic agent [21].

The EOs of *J. phoenicea* origin are typically rich in monoterpenoids and their derivatives [22], although their specific chemical constituents vary significantly according to the plant organ, phenological stage, and geographical distribution, among other factors. Distinct authors have previously reported the chemical composition of EOs of *J. phoenicea* collected from several origins such as Algeria, France, Morocco, Greece, Jordan, Egypt, and Tunisia. Importantly, besides the chemical composition, most studies have exploited the EOs antioxidant and antimicrobial potential [2,17,23,24,25,26,27]. In the case of Tunisian plants, these investigations used EOs derived from the leaves and/or fruits of plants growing in the southern [28,29] and central [17] regions of the country. However, the chemical composition of *J. phoenicea* grown in the north of Tunisia has not been studied. Moreover, in contrast to the screening of *J. phoenicea* EOs’ antioxidant and antibacterial capabilities, data on the species’ anticancer and anti-tyrosinase actions are extremely scarce and limited to Algerian and Libyan species [24,30]. Thus, this study’s primary goals were to investigate the EOs’ cytotoxic effects on cancer cells and their anti-tyrosinase properties, while also assessing the chemical composition of EOs from leaves and berries of *J. phoenicea* from the north of Tunisia for the first time.

## 2. Results and Discussion

### 2.1. Yield and Composition of the Essential Oils

The EOs of *J. phoenicea* L. leaves and berries represented 1.69 and 0.45% of the dry plant mass, respectively (Table 1). The EO obtained from the leaves was significantly more representative than that reported by Aouadi et al. [17] and Ennajar et al. [31], obtained from Tunisian *J. phoenicea* harvested from localities in the center west and the southeastern area of Tunisia (0.9 and 0.8%, respectively), whereas that from berries was less representative than those obtained from fruits collected from different Tunisian localities [29]. The yield of EO from leaves was also higher than those reported by other authors for *J. phoenicea* leaves from Morocco, Algeria, Greece, and Spain (0.21 to 1.2%) [32,33,34], while the EO from berries was less representative than that obtained from Egyptian fruits (1.96%) [24]. Obviously, these variations can be attributed to genetic and environmental factors, as well as various harvesting locations and plant development stages. In fact, soil type and environmental conditions like precipitation, temperature, and solar radiation may influence the essential oil yield [35].

**Figure 1:**
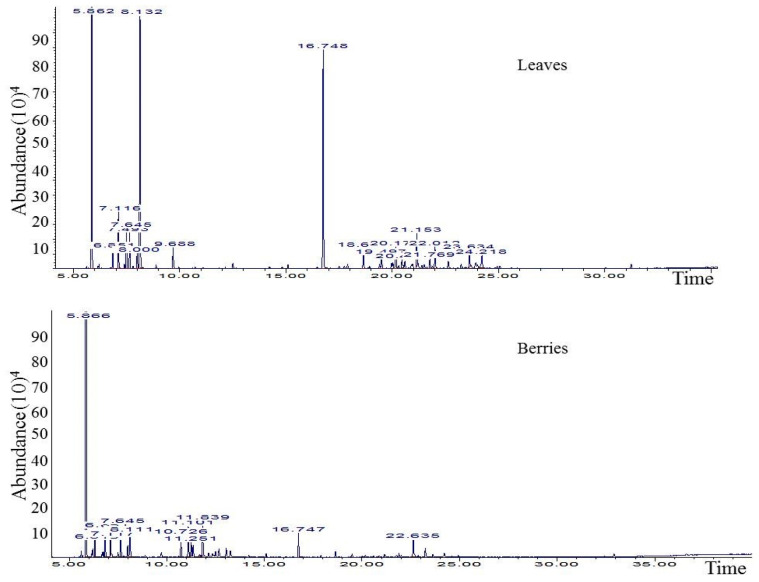
GC-MS chromatogram of *Juniperus phoenicia* leaves and berries essential oils.

The GC/MS data from leaves and berries EOs are presented in Table 1/Figure 1. Overall, both EOs were characterized by the prevalence of terpenic hydrocarbons (88% in leaves and 92% in berries), which, as expected, was mostly represented by α-pinene (levels of 44 and 84% in leaves and berries, respectively). In fact, α-pinene has been highlighted as a marker compound for *J. phoenicea* in previous studies, with levels varying among origin. In this respect, EO obtained from *J. phoenicea* fresh leaves collected from Spain and Greece exhibited α-pinene at levels that varied between 42 and 54% [32], while in others, such as that obtained from Morocco plants, α-pinene represented more than 64% [2,37,38,39]. Consistent with these studies, all the available literature data focused on EOs from leaves and/or fruits of *J. phoenicea* of Tunisia (mid-west and south parts of the country) had the predominancy of monoterpenes, particularly α-pinene [17,25,26,27].

Although leaves and berries exhibited the same EO chemotype, these presented noticeable chemical differences: the EO of berries was characterized by the presence of oxygenated monoterpenes at a level of 7%, mostly represented by trans-verbenol (2%) and trans-*p*-menth-2-ene-1,8-diol (2.5%), which were absent in the EO obtained from the leaves. In turn, this latter showed high levels of other monoterpenes like β-phellandrene (18%) and camphene (15%), followed by sesquiterpenes, which included trans-caryophyllene, germancrene-D, δ-cadinene and γ-elemene. Of note, the herein-registered levels of β-phellandrene and camphene in the leaves EO were much higher than those previously reported by other authors for plant leaves from *J. phoenicea* of Tunisian origin (Matmata and Sousse regions) [31,40]. Still, β-phellandrene was found at 21-25% in leaves EO of *J. phoenicea* collected from Spain and Corsica [32,41]. Such variability on terpene biosynthesis is expected to occur due to genetic and/or environmental factors [42,43,44].

### 2.2. Cytotoxic Activity of J. phoenicia L. Essential Oils

**Figure 2:**
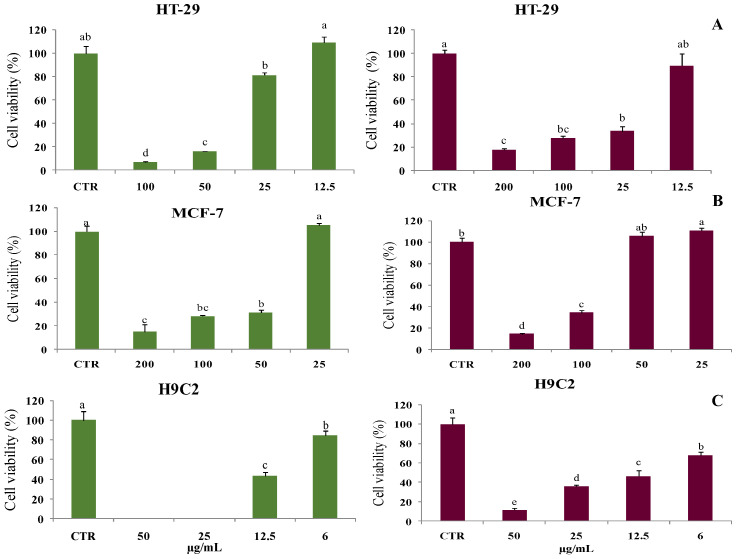
Cell viability using resazurin assay in the colon HT-29 cell line (**A**), in breast MCF-7 cell line (**B**) or in cardiomyoblasts H9C2 (**C**) in the presence of *J. phoenicia* leaves and berries EOs (at the left in green and at the right, in red, respectively), for 48 h. Data are represented as the mean ± standard deviation. Different superscript letters (a, b, c, d, e) represent significant differences at *p* < 0.05 according to Duncan’s test.

The cytotoxicity potential of leaves and berries EOs was evaluated against two cancer cell lines, MCF-7 and HT-29, and against normal human cardiomyoblasts cells H9C2. The results showed that MCF-7 and HT-29 carcinomas proliferation were strongly inhibited by the two EOs after 48 h of treatment, in a dose-dependent manner (Figure 2).

The IC_50_ values of the cytotoxic activity of *J. phoenicia* leaves and berries EOs against the examined breast and colon cancer cells ranged between 15 and 60 µg/mL (Table 2). It is noticeable that EO of leaves had a strong cytotoxic effect against the two cancer cell lines (IC_50_ values of 38–40 μg/mL), whereas the berry’ EO was more effective against the HT-29 cell line (IC_50_ of 15 ± 0.43 µg/mL). On the other hand, both EOs were found to be cytotoxic against H9C2 myoblast cells (IC_50_ = 12 µg/mL). Note that this cell line is typically used to follow processes of myocyte damage, assessment of toxic effects of studied compounds on apoptosis and necrosis in cardiac myocytes, and cardiotoxicity analyses of novel medications mostly anticancer drugs [44].

**Figure 3:**
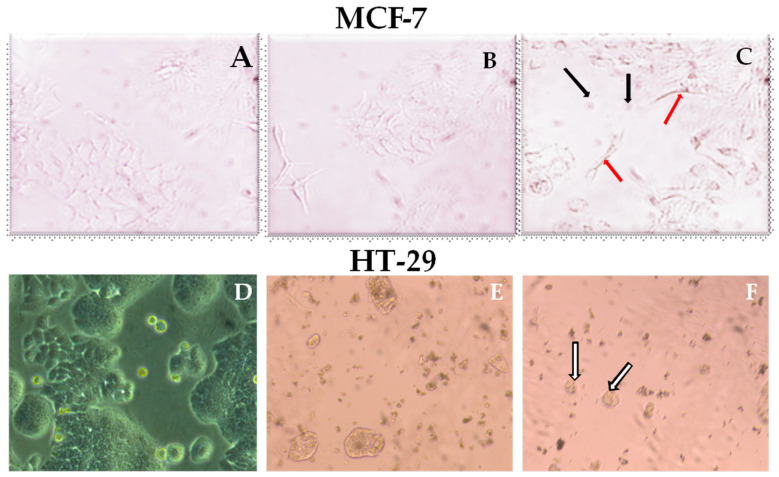
Inverted microscopy photo of MCF-7 and HT-29 cancer cells after 48 h incubation: (**A**,**D**) MCF-7 and HT-29 cancer cells in the absence of essential oil (untreated cells); (**B**) MCF-7 cancer cells treated with leaves EO at 100 µg/mL; (**C**) MCF-7 cells treated with berry EO at 50 µg/mL; (**E**) HT-29 cancer cells in contact with leaf EO at 100 µg/mL; (**F**) HT-29 cancer cells in contact with berry EO at 25 µg/mL. Black arrow indicates cell shrinkage and condensation, red arrow indicates echinoid spikes, and white arrow indicates apoptotic bodies.

The cytotoxic effect of *J. phoenicea* EOs on cancer cells was examined by phase contrast microscope (Figure 3). The treatment of MCF-7 cells with berries EO at 50 µg/mL caused visible morphological changes, including cell shrinkage, a decrease in cell number, chromosomal and cytoplasmic condensation, the formation of cytoplasmic blebs and echinoid spikes, in opposition to the tightly packed and distinctively epithelial monolayer formation in the untreated cells. Likewise, the treatment of HT-29 cancer cells with 25 µg/mL oil induced a visible decrement of cell number while 100 µg/mL of leaves EO induced a notable cytotoxic effect with formation of apoptotic bodies. These findings suggest that *J. phoenicea* EO from berries and leaves effectively suppress the cell proliferation in MCF-7 and HT-29 cell lines through apoptosis.

Our results demonstrated that the EOs of berries and leaves had significant anticancer activity against the examined breast and colon cancer cells. Studies investigating the anticancer activity of *J. phoenicia* EOs are very scarce. Cheraif et al. [30] tested the cytotoxicity of *J. phoenicia* leaves EOs collected from Algeria against breast cancer and reported lower activity that than obtained in our study, with an IC_50_ value of 320 µg/mL. In turn, leaves and berries EOs of *J. phoenicia* from Libya showed high activities against tumor cell lines including brain, lung, liver, and breast carcinoma cell lines, with IC_50_ varying from 0.6 to 5 µg/mL [24]. Interestingly, other Juniperus species exhibited anticancer activity. Among them, the EO of *Juniperus macrocarpa* was shown to inhibit the cell proliferation of MCF-7, with an IC_50_ value of 85.4 μg/mL [45]. Moreover, the EOs isolated from the leaves, seeds and fruits of *Juniperus oxycedrus* were reported to be active against human breast adenocarcinoma (MCF-7), human chronic myelogenous leukemia (K562) and a human neuroblastoma cell line derived from a highly malignant tumor (SHSY5Y) cells [46].

The interesting cytotoxic activity of *J. phoenicia* EO of leaves and berries recorded in our study could be due to their richness in α-pinene, since this monoterpenes claimed to be a potent cytotoxic agent against different breast cancer cell lines such as MDA-MB-468/231 and MCF-7, as well as against UACC-257 melanoma cells [47], HeLa cervical carcinoma cells [48], A549 lung cancer cells, HepG2 hepatocellular carcinomic cells [49,50], and the human ovarian cancer cell lines SK-OV-3 and HO-8910 [51]. α-Pinene induces apoptotic cell death via caspase activation in human ovarian (PA-1) cancer cells [52]. Additionally, it was observed that HepG2 cells treated with α-pinene exhibited growth inhibition and cell cycle arrest [53].

In addition, leaves EO was rich in camphene (15%, Table 1), which is also claimed to display antitumor activity in vivo, as well as to induce intrinsic apoptosis in melanoma cells [54]. Moreover, leaves were characterized by high levels of sesquiterpenes (9.6%) while bisabolene was present at low level (1.4%) in berries. The high potential of these compounds and their derivatives against various cancers like breast and colon has been reported, even when present at low percentages [55].

### 2.3. Tyrosinase Inhibition Activities of J. phoenicea EOs

Pigmentation disorders, identified as the restricted or uneven distribution of melanin pigment, are presently one of the chief targets of modern cosmetics or dermatological treatments. Tyrosinase (EC.1.14.18.1), a copper enzyme of the melanogenesis process (melanin biosynthetic pathway), is a primary target of skin-lightening substances. This enzyme catalyzes the hydroxylation of L-tyrosine to L-dihydroxyphenyl alanine (DOPA), via its monophenolase activity and its further oxidation via its diphenolase activity to the corresponding *O*-quinone, the dopaquinone, which polymerizes spontaneously to form melanin [56]. Briefly, tyrosinase is involved in the first steps of melanin biosynthesis inside the melanocytes [30].

EOs are known for their ability to inhibit tyrosinase due their complexity and chemical composition [57], owing to a synergistic interaction of their compounds with the enzyme. The ability of *J. phoenicea* EOs to restrain tyrosinase was assessed in terms of its two catalytic activities, i.e., monophenolase and diphenolase activities, and compared to the inhibitory capacity of kojic-acid, which is recognized to inhibit tyrosinase (positive control). As can be observed in Table 3, the two EOs exhibited distinct potencies in this regard, being much more active towards diphenolase activity, compared to monophenolase. Interestingly, the berries EO was more potent than that of leaves, possessing half of potency of kojic acid (IC_50_ value of 109 μg/mL vs. 52 μg/mL, respectively).

There are very few studies in the literature that explored the *J. phoenicia* EO tyrosinase inhibitory activity. A recent study conducted by Cheraif et al. [30] showed *J. phoenicia* leaves EO from Algeria possessed low tyrosinase inhibitory activity (11% in the presence of 1 mg/mL of EO, when using DOPA as substrate). The high inhibitory potential of *J. phoenicia* EOs obtained in our study may be associated with some specific compounds. In fact, Ho et al. [58] reported that *Alpinia speciosa* seed EO that contain camphor, terpinen-4-ol and α-pinene as major compounds, was a good tyrosinase inhibitor. Additionally, myrcene was previously reported to exhibit strong tyrosinase inhibition [59]. On the other hand, β-caryophyllene was shown to reduce melanogenesis in murine B16F10 cells by down-regulation of tyrosinase expression [60]. In a previous work performed with *J. phoenicea* berry EO, rich in α-pinene, the authors proved it to possess anti-aging potential in in vitro and in vivo models [61].

Overall, the differing inhibitory capacity towards monophenolase and diphenolase activities observed in our case for *J. phoenicia* EOs is likely due to the different mechanisms of tyrosinase inhibition. Reducing substances may lead to the chemical reduction in dopaquinone, which prevents the synthesis of dopachrome and melanin by reducing back *O*-dopaquinone to DOPA. In turn, *O*-Dopaquinone scavengers, which are well-known melanogenesis inhibitors and react with dopaquinone to produce colorless products, include most thiol-containing compounds. As a result, the melanogenesis process is slowed until all the scavenger is spent, at which point it resumes its previous pace [62].

To deduce their relationship, correlation plots between the major identified compounds in both leaves and berries, i.e., α-pinene, β-phellandrene and camphene, with the IC_50_ values of monophenolase and diphenolase activities were applied, and the respective correlation coefficients are summarized in Table 4. This analysis resulted in a high correlation factor (r > 0.8) between those compounds and their inhibitory effects towards monophenolase and diphenolase activities, which highlights the potent role of monoterpene compounds in melanin synthesis inhibition. Consistent with this, the major compounds of *Syzygiumsa marangense* (Blume) EO, α-pinene and β-pinene, were found to radically restrict tyrosinase activity, in addition to anti-BChE properties. The co-treatment with α-pinene from *Melaleuca quinquener* via (Cav.) EO (MQ-EO) decreased tyrosinase activity in α-melanocyte-stimulating hormone (α-MSH)-stimulated B16 melanoma cells in a dose-dependent manner. MQ-EO at a concentration of 20 μg/mL powerfully diminished tyrosinase activity in the α-MSH-stimulated cells and illustrated the uppermost inhibitory effects on melanin production and tyrosinase activity [63].

### 2.4. Antioxidant Activity of J. phoenicia L. Essential Oils

EOs of *J. phoenicia* leaves and berries were tested for their antioxidant capacity using DPPH radical scavenging assay and ferric reducing antioxidant power (FRAP). According to the results of the DPPH test and FRAP illustrated in Table 5, the EOs exhibited only moderate scavenging activity. Still, among the two EOs, that from leaves was more active (IC_50_ value of 11.5 ± 0.04 mg/mL and vs. 14.5 ± 1.67 mg/mL). Our findings are in line with recent studies that reported moderate antiradical activity for *J. phoenicia* EOs [28,31,34,64].

The antioxidant capacities of *J. phoenicia* EOs could be associated with monoterpene hydrocarbons and oxygenated monoterpenes, including α-pinene [65]. In this regard, De Christo Scherer et al. [66] showed that terpinolene and α-phellandrene possess the ability to scavenge reactive species. Moreover, when investigating the cytoprotective mechanisms of verbenol in in vivo and in vitro ischemia models, In-Young et al. demonstrated its antioxidant ability by reducing the intracellular level of reactive oxygen species [67].

Previous studies reported that EOs are natural sources of antioxidants with numerous modes of action, such as free radical scavenging, prevention of chain initiation, termination of peroxides and reducing agents. The activity of EO may result from the interaction, including synergy, antagonism, and/or additive effects, of volatile compound mixtures [30].

## 3. Materials and Methods

### 3.1. Plant Material and Essential Oil Extraction

*Juniperus phoenicea* L. leaves and berries were harvested in May 2022 at Boukornine mountain from Ben Arous governorate (24 km from capital of Tunis; 36°42′18″ N and 10°20′00″ E; sub-humid to mild winter; mean annual rainfall: 450–550 mm), during the flowering period. In general, the berries of this species resemble galbulus, are about 1 cm across, and are dark brown to red in color, containing 3–9 seeds. The leaves are decussate scales, alternating in pairs or trios, oval to rhombic, green–blue-green in color, and 1–2 mm long.

**Figure 4:**
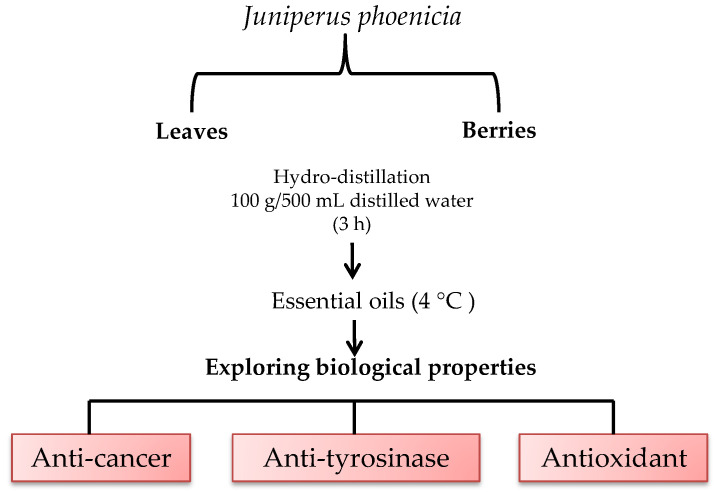
Graphical scheme of study approach.

Plants were identified by the botanist of the Biotechnology Center of Borj-Cedria (CBBC), and a voucher specimen [F-RE 30] was deposited at the Herbarium of the Laboratory of Aromatic and Medicinal Plants (LPAM). Berries and leaves were collected and dried at room temperature in the absence of direct light. A measure of 100 g of each sample (leaves/berries) was subjected to hydrodistillation for 3 h with 500 mL distilled water using a Clevenger-type apparatus. The obtained essential oil was collected and dried over anhydrous sodium sulfate and stored in sealed glass vials in a refrigerator at 4 °C prior to analysis (Figure 4). The yield of berry and leaf EOs was calculated on the dried weight basis.

### 3.2. Gas Chromatography–Mass Spectrometry (GC-MS) Analysis

The analysis of the volatile constituents was run on a Hewlett-Packard GC-MS system (GC: 5890-series II; MSD 5972, Palo Alto, CA, USA), equipped by a fused-silica HP injector port and detector were, respectively, at 250 °C and 280 °C and the split ratio was 1/50 as previously described [68]. The software adopted to handle mass spectra and chromatograms was a Chem Station. All constituents were identified by comparison of their Kovats retention indexes with those in the literature [36]. The calculation of the Kovats index was made based on a linear interpolation of the retention time of the homologous series of n-alkanes (C8-C22) under the same operating conditions. The components were also identified by comparing the mass spectra of each constituent with those stored in the Wiley 275 GC-MS and FFNSC1.3 libraries and with mass spectra from the literature [36]. Quantification of *J. phoenicia* berry and leaf EO constituents was determined after normalizing the areas of each detected compound and expressed as a percentage of total area (%).

### 3.3. Anti-Proliferative Effect by Resazurin Assay

The hormone-dependent human MCF-7 breast cancer cell, HT-29 colon cancer and the normal cells H9C2 cardiomyoblasts were obtained from ATCC and cultured at 37 °C in humidified 5% CO_2_ atmosphere in Dulbecco’s modified Eagle’s medium (Sigma, (St. Louis, MO, USA)), supplemented with 10% heat inactivated fetal bovine serum (Gibco-BRL, Paisley, Renfrewshire, Scotland) and antibiotics (100 mg/mL streptomycin and 194 U/mL penicillin). MCF-7, HT-29 and H9C2 (2 × 10^4^ cells/mL) cells were cultured in 96-well during 24 h, following treatment with leaf/berry *J. phoenicia* EOs at different concentrations. The EOs were dissolved in DMSO and then diluted with the culture medium into different concentrations (200, 100, 50, 25, 12.5, and 6 μg/mL) to make the final DMSO concentration at less than 0.5% (*v*/*v*), in order to avoid solvent toxicity. After 24 h of treatment, the fluorescence was determined using an automated 96-well Fluoroskan Ascent FlTM plate reader (Thermo-Labsystems, Waltham, MA, USA) at an excitation wavelength of 530 nm and an emission wavelength of 590 nm. For all assays, cytotoxic activity was expressed as the percentage of cell viability. All samples were analyzed in triplicate.

### 3.4. Determination of Tyrosinase Inhibition Effects

Tyrosinase inhibition activity was determined as described by Momtaz et al. [69], with L-3,4-dihydroxyphenylalanine (L-DOPA) or L-tyrosine as substrates. Samples were dissolved in DMSO, and further diluted in potassium phosphate buffer (50 mM, pH 6.5). Assays were carried out in a 96-well plate and absorbance was read on a Multiskan FC microplate reader (Thermo scientific technologies, Shanghai, China). Each oil (70 μL) was mixed with 30 μL of tyrosinase (333 units/mL in phosphate buffer, pH 6.5). After 5 min of incubation at room temperature, 110 μL of substrate (2 mM L-tyrosine or 12 mM L-DOPA) were added and the reaction mixture was further incubated for 30 min. Kojic acid was used as a positive control, and a blank contained all the components except L-tyrosine or L-DOPA. Absorbance was measured at 492 nm, and the percentage of tyrosinase inhibition was calculated as follows:

Tyrosinase inhibition (%) = [(A_control_ − A_sample_)/A_control_] × 100,

where A_control_ and A_sample_ are the absorbances of the blank and of the test reaction mixture (containing oil or kojic acid), respectively. The IC_50_ values of extracts and kojic acid were calculated from linear regression curve and expressed in μg/mL.

### 3.5. Evaluation of Antioxidant Capacities of J. phoenicia EO

#### 3.5.1. DPPH Scavenging Assay

DPPH quenching ability of *J. phoenicia* EOs was measured as previously reported [19]. Standards of butylhydroxytoluene (BHT), vitamin C, and α-tocopherol were separately used. Briefly, 250 µL of methanolic solution of stable radical DPPH (0.2 mM) was added to 1000 µL of increasing concentrations of *J. phoenicia* EOs. After 30 min of incubation at room temperature, the absorbance was read against a blank at 517 nm. DPPH scavenging ability was expressed as IC_50_ (mg mL^−1^) which is the inhibiting concentration for 50% inhibition. The inhibition percentage (IP %) of DPPH radical was calculated using the following formula:

IP (%) = [(A_control_ − A_sample_)/A_control_] × 100

#### 3.5.2. Ferric Reducing Antioxidant Power (FRAP) Assay

This test, based on the reduction in the trivalent iron produced by the FeCl_3_, was performed according to a previous method [70]. The intensity of the blue-green color was measured at 700 nm and values were expressed as EC_50_ (mg/mL), i.e., the effective concentration of *J. phoenicia* berry and leaf EOs corresponding to an OD = 0.5.

### 3.6. Statistical Analysis

Data were subjected to one-way analysis of variance for means of comparison, and significant differences were calculated according to Duncan’s multiple range test. Data are reported as means ± standard error of the means. Differences at *p* < 0.05 were considered statistically significant. SPSS (version 11.0) was used to perform the statistical analysis.

## 4. Conclusions

This research promotes the use of *J. phoenicia* leaves and berries EO as a platform for drug development as it may serve as an excellent lead for the development of agents for breast and colon cancers. Leaves EO possess remarkable cytotoxic property on MCF-7 while berries EO showed potential cytotoxic effects on HT-29 cancer cell lines. The two oils, and in particular the berries’ EOs, exhibited high tyrosinase inhibition rate and interesting antioxidant activity. These oils should, therefore, be re-considered as sources of bioactive compounds with potential applications ranging from food preservation, given their antioxidant power, to cosmetic care formulations such nano-emulsion technology, in view of the higher market trend for nano-cosmeceutical products containing primarily naturally extract compounds, replacing synthetic chemicals, which meets consumers’ growing awareness of safer products for use on the skin. To this end, more research is needed to improve the nanotechnology of plant-based nano-cosmetics, thereby ensuring that nano-scaled plant extract or essential oil-loaded formulation remains the best and safest choice in the coming years.

## Figures and Tables

**Table 1:** Chemical composition (%, *w*/*w*) of essential oils from *Juniperus phoenicea* L. leaves and berries.

			Relative Percentage	
Compounds	KI ^a^	KI ^b^	Leaves	Berries
α-Pinene	940	939	44.2	83.6
Verbenene	975	967	-	0.8
β-Pinene	981	980	1.1	1.4
β-Myrcene	988	991	3.1	0.9
α-Phellandrene	1005	1005	2.1	-
δ-3-Carene	1011	1011	2.4	1.7
o-Cymene	1028	1026	0.9	-
Limonene	1032	1031	-	1.7
β-Phellandrene	1031	1031	18.0	-
α-Terpinolene	1089	1088	1.5	-
Campholene aldehyde	1025	1025	-	1.3
Trans-verbenol	1144	1144	-	2.1
Camphor	1145	1143	-	1.0
Trans-p-Menth-2-Ene-1,8-Diol	1340	1344	-	2.5
Camphene	954	953	15.0	1.5
Trans-caryophyllene	1420	1418	1.5	-
β-Selinene	1419	1419	0.9	-
Germacrene-D	1480	1481	1.5	-
(+)-epi-Bicyclosesquiphellandrene	1482	1482	0.8	-
δ-cadinene	1525	1524	2.2	-
Elemol	1547	1549	0.7	-
γ-Elemene	1429	1429	1.5	-
α-Longipinene	1345	1347	1.2	-
β-Bisabolene	1507	1509	-	1.4
α-Cadinol	1653	1653	1.3	-
Monoterpenes hydrocarbons			88.3	91.6
Oxygenated monoterpenes			-	6.9
Sesquiterpenes hydrocarbons			9.6	1.4
Oxygenated sesquiterpenes			2.1	-
Yield			1.69	0.45

Compounds tentatively identified using authentic standard; “-”: not detected. KI: Kovat’s index. KI ^a^: experimental Kovat index. KI ^b^: Kovat index from the literature [34,36].

**Table 2:** Cytotoxic effect (IC_50_, μg/mL) of *Juniperus phoenicea* L. leaves and berries essential oils.

Cell Type	Leaves	Berries
HT-29	38 ± 0.98 ^a^	15 ± 0.43 ^b^
MCF-7	40 ± 1.22 ^b^	60 ± 2.14 ^a^
H9C2	12 ± 0.77 ^a^	12 ± 0.93 ^a^

Data are represented as the mean ± standard deviation. Different superscript letters (a, b) in the same line represent significant differences at *p* < 0.05 according to Duncan’s test.

**Table 3:** Antityrosinase activity of *Juniperus phoenicea* L. leaves and berries essential oils.

Samples	Monophenolase (μg/mL)	Diphenolase (μg/mL)
Leaves	944 ± 0.22 ^a^	371 ± 0.42 ^a^
Berries	455 ± 1.19 ^b^	109 ± 0.74 ^b^
Kojic-acid	7.06 ± 0.24	52.01 ± 1.98

Data are represented as the mean ± standard deviation. Different superscript letters (a, b) in the same column represent significant differences at *p* < 0.05 according to Duncan’s test.

**Table 4:** Correlation coefficients between α-pinene, β-phellandrene, and camphene with the IC_50_ values of anti-proliferative or monophenolase and diphenolase activities.

Variables	Monophenolase	Diphenolase	α-Pinene	β-Phellandrene	Camphene
Monophenolase	1	1	−0.91	−0.94	−0.99
Diphenolase	1	1	−0.87	−1	−0.96
α-Pinene	−0.91	−0.87	1	0.74	0.99
β-Phellandrene	−0.94	−1	0.74	1	0.81
Camphene	−0.99	−0.96	0.99	0.81	1

**Table 5:** Antioxidant activity of *J. phoenicea* L. leaves and berries essential oils.

Samples	DPPH Test IC_50_	FRAP Test EC_50_
Leaf (mg/mL)	11.5 ± 0.04 ^b^	14.5 ± 1.67 ^b^
Berry (mg/mL)	14.0 ± 3.25 ^a^	17.0 ± 0.96 ^a^
BHT (µg/mL) *	19.5 ± 1.33 *	ND
Vitamin C (µg/mL) *	14.5 ± 0.52 *	28.3 ± 0.98 *
α-Tocopherol (µg/mL) *	17.8 ± 1.78 *	22.5 ± 3.77 *

ND—not determined. * µg/mL. Data are represented as the mean ± standard deviation. Different superscript letters (a, b) in the same column represent significant differences at *p* < 0.05 according to Duncan’s test.

## Data Availability

Data are contained within the article.
